# MIDcor, an R-program for deciphering mass interferences in mass spectra of metabolites enriched in stable isotopes

**DOI:** 10.1186/s12859-017-1513-3

**Published:** 2017-02-03

**Authors:** Vitaly A. Selivanov, Adrián Benito, Anibal Miranda, Esther Aguilar, Ibrahim Halil Polat, Josep J. Centelles, Anusha Jayaraman, Paul W. N. Lee, Silvia Marin, Marta Cascante

**Affiliations:** 1 Department of Biochemistry and Molecular Biology, Faculty of Biology, Universitat de Barcelona, Barcelona, 08028 Spain; 2 Institute of Biomedicine of the Universitat de Barcelona (IBUB) and Associated Unit to CSIC, Barcelona, Spain; 3 Department of Pediatrics, Harbor-UCLA Medical Center, Research and Education Institute, Torrance, CA 90502 USA

**Keywords:** ^13^C labeling of metabolites, Gas chromatography/mass spectrometry, Correction of peaks overlapping, Isotopic effect, Computational analysis, R-program

## Abstract

**Background:**

Tracing stable isotopes, such as ^13^C using various mass spectrometry (MS) methods provides a valuable information necessary for the study of biochemical processes in cells. However, extracting such information requires special care, such as a correction for naturally occurring isotopes, or overlapping mass spectra of various components of the cell culture medium. Developing a method for a correction of overlapping peaks is the primary objective of this study.

**Results:**

Our computer program-MIDcor (free at https://github.com/seliv55/mid_correct) written in the R programming language, corrects the raw MS spectra both for the naturally occurring isotopes and for the overlapping of peaks corresponding to various substances. To this end, the mass spectra of unlabeled metabolites measured in two media are necessary: in a minimal medium containing only derivatized metabolites and chemicals for derivatization, and in a complete cell incubated medium. The MIDcor program calculates the difference (**D**) between the theoretical and experimentally measured spectra of metabolites containing only the naturally occurring isotopes. The result of comparison of **D** in the two media determines a way of deciphering the true spectra. (1) If **D** in the complete medium is greater than that in the minimal medium in at least one peak, then unchanged **D** is subtracted from the raw spectra of the labeled metabolite. (2) If **D** does not depend on the medium, then the spectrum probably overlaps with a derivatized fragment of the same metabolite, and **D** is modified proportionally to the metabolite labeling. The program automatically reaches a decision regarding the way of correction. For some metabolites/fragments in the case (2) **D** was found to decrease when the tested substance was ^13^C labeled, and this isotopic effect also can be corrected automatically, if the user provides a measured spectrum of the substance in which the ^13^C labeling is known a priori.

**Conclusion:**

Using the developed program improves the reliability of stable isotope tracer data analysis.

**Electronic supplementary material:**

The online version of this article (doi:10.1186/s12859-017-1513-3) contains supplementary material, which is available to authorized users.

## Background

Metabolic flux analysis based on the incorporation of ^13^C, originated from artificially enriching ^13^C into the metabolites of central carbohydrate metabolism, is now a widely applied method of investigation providing access to the regulation of metabolism in living cells [1–9]. Gas chromatography coupled to mass spectrometry (GC/MS) is a basic technique used for monitoring ^13^C isotopic isomers (isotopomers) that are different in their mass number (mass isotopomers). In October 2012, the European COordination of Standards in MetabOlomicS (COSMOS) consortium, comprising 14 European partners, started its work on metabolomics data standardization, publication and dissemination workflows. The algorithms presented here, prepared in the framework of this project, were developed with the objective to provide a growing database i) with reliable curated data and ii) computer tools for the initial step of data analysis.

The artificial ^13^C labels cannot be distinguished from various isotopes occurring in the environment and recorded by GC/MS as an increase of the mass of the analyzed molecules. These molecules are the chemical derivates of metabolites created by chemical binding of the metabolite molecules to other reagents to produce a form suitable for GC/MS recording (see Methods section for details). Electronic ionization applied for GC/MS analysis may result in splitting the derivates into fragments that may contain fragments of the metabolites designated for testing. The measured mass isotopomer distribution (MID) should be corrected for the naturally occurring isotopes to reveal the distribution of only the artificial ^13^C labels, which is necessary for subsequent fluxomic analysis. Methods of the correction for the naturally occurring isotopes ^13^C and ^15^N in an analyzed metabolite were first developed by Brauman [10], based on calculations of the theoretical natural MID using the observed frequencies of the isotopes in the environment and chemical compositions of assayed molecules. Various modifications of this method were developed, e.g., [11–14]. Some derivates contain Si, which in addition to the most abundant isitope ^28^Si, has substantial fractions of ^29^Si and ^30^Si [15, 16]. Recently published algorithms [17] allow mass spectra of derivatized metabolites to be easily corrected for naturally occurring isotopes such as ^57^Fe and ^77^Se. However, despite the well-developed correction schemes for naturally occurring isotopes, the correction of raw MID data still needs improvements. The measured MID can differ from the corresponding calculations that account for the presence of naturally occurring isotopes. As summarized in [17], the existing algorithms treat the observed difference formally as noise, not considering its causes. The objective of presenting here an algorithm for raw MID data correction is to provide a tool, which, in addition to correcting for natural isotope occurrence, corrects the data in cases where mass peaks in a mass spectrum overlap with those for other metabolites. Overlapping MS signals for more than one metabolite is an important cause of differences between experimentally measured and corresponding MID calculated theoretically. We distinguish two such an overlapping cases: either 1) with patterns of unlabeled molecules, which depends only on the composition of the assay medium, or 2) with another pattern of mass isotopomers of the same molecules designated for testing, which depends on their artificial labeling. Our open source software MIDcor (https://github.com/seliv55/mid_correct), developed in the R programming language, uses the previously described methods for separation of natural and artificial labeling [10, 11, 13], and, also, corrects the peaks overlapping either with unlabeled or labeled metabolites.

## Methods

### Experimental

#### Cell culture

Human immortalized fibroblasts BJ (ATCC, Germany) were cultured in media consisted of DMEM (Gibco) and Medium 199 (Sigma-Aldrich) in 4:1 proportion containing 10% FBS (AG Biochrom), 10 mM glucose, 3 mM glutamine, 1 mM pyruvate, 0.085 mg/mL hygromycin B (Roche), 0.4 mg/mL puromycin (Sigma-Aldrich) and 1% antibiotic. In our experiments the passage number never exceeded 10.

#### Labeling of metabolites with ^13^C

Cells were incubated with the tracer-containing medium (either 10 mM, 50% enriched [1,2-^13^C2]-glucose or 2 mM, 100% enriched U-^13^C-glutamine) for 8 and 24 h. At the end of incubations, the media were collected and frozen for glucose and amino acids analysis. For the analysis of intracellular metabolites, dishes were frozen at -80 °C until starting the analysis.

#### GC-MS and MID analysis

##### Cell culture medium

Glucose was extracted using ion exchange chromatography and derivatized to its aldonitrile acetate form [18]. We monitored the ion cluster around the m/z 328 (carbons 1–6 of glucose, chemical ionization) to find the molar enrichment of ^13^C. Lactate was extracted and derivatized to its propylamideheptafluorobutyric form [19, 20]. The m/z 328 (carbons 1–3 of lactate, chemical ionization) was monitored. The amino acids were extracted using ion exchange chromatography, derivatized to their n-trifluoroacetyl-n-butyl ester forms as is [21]. The ion clusters around m/z 152 and 198 (carbons 2–4 and 2–5 of glutamate, respectively, electron impact ionization), m/z 228 (carbons 1–2 of glycine, chemical ionization) and m/z 354 (carbons 1–3 of serine, chemical ionization) were monitored.

##### Intracellular metabolites

Cells were scraped using methanol-water. An equivalent volume of chloroform was then added, and the aqueous phase was collected and evaporated under airflow for polar intracellular metabolite analysis. After dissolution in 50 μL of 2% methoxyamine hydrochloride in pyridine, the tert-butylmethylsilyl derivative was prepared by adding 30 μL of N-methyl-N-(tert-butyldimethylsilyl) trifluoroacetamide (MBTSTFA) + 1% tert-butyldimethylchlorosilane (TBDMCS; Sigma) and incubating at 55 °C for 1 h [22]. We monitored the ion clusters around m/z 459 (carbons 1-6 of citrate, electron impact ionization), m/z 174 (carbons 1–3 of pyruvate, electron impact ionization) and m/z 418 (carbons 1–4 of aspartate, electron impact ionization).

Mass spectral data were obtained on a 7890A mass spectrometer coupled with a 5675C gas chromatograph (Agilent Technologies). The settings are as follows: GC inlet 230 °C, transfer line 280 °C, MS source 230 °C, MS quad 150 °C. An HP-5 capillary column (30 m length, 250 μm diameter, 0.25 μm film thickness) was used for analysis of all metabolites.

### Theoretical aspects

#### Calculation of natural ^13^C distribution

The correction for naturally occurring isotopes requires calculation of the theoretical MID based on the observed isotope occurrence in the environment and the chemical composition of the analyzed derivate of the tested metabolite. The algorithm for calculating the natural isotope distribution, provided in the MIDcor program, uses commonly accepted method [10–17]. Additional file 1 Text S1 provides the details.

#### Correction of the H+ loss provoked by electron impact

Although the molecules combined from the isotopes with smallest mass are expected to give the lightest mass isotopomers (designated as M), it is normal that a peak corresponding to the mass of M-1 is registered [13, 23]. Table 1, which provides an example of raw data obtained with GC/MS, illustrates such a peak.

**Table 1:** The intensities GC/MS peaks for cold TMS-derivative of aspartate fragment

m/z:	417(M-1)	418(M)	420(M + 1)	420(M + 2)	421(M + 3)	422(M + 4)	423(M + 5)
Sample 1	704	112249	40291	19821	4202	997	97
Sample 2	713	104212	37495	18416	3657	909	47

The isotopomers of M-1 can appear due to H+ loss as a side effect of an impact of the electron flow used for ionization of molecules in a GC/MS apparatus. This effect could result in a systematic error in the experimental determination of the MID, and the MIDcor program corrects it. In the example, provided in Table 1, the fraction of M-1 is 0.7% of the peak corresponding to M. The same portion of H+ loss is assumed for all other mass isotopomers. This effect decreases the weight of isotopomers and thus shifts them from their proper peak in the MS recording to a position one mass unit less.

A correction for “shifted” isotopomers, implemented in the MIDcor program, is based on the calculation of the ratio of isotopomers (M-1) to (M) in a commercial preparation of unlabeled metabolite:

1 $$ \mathrm{f} = {\mathrm{N}}_{\left(\mathrm{M}\hbox{-} 1\right)}/{\mathrm{N}}_{\mathrm{M}} $$

This factor (f), reflecting the portion of isotopomers shifted due to H+ loss, should be applied to all peaks to return the corresponding amounts from (M + i-1) to (M + i):

2 $$ {N}_{\left(\mathrm{M}+\mathrm{i}\right)}^{\mathrm{corr}}={\mathrm{N}}_{\left(\mathrm{M}+\mathrm{i}\right)}^m\cdot \left(1+\mathrm{f}\right)-{N}_{\left(\mathrm{M}+\mathrm{i}+1\right)}^m\cdot f $$

Here “m” stand for “measured” and “corr” for “corrected”. After such a correction the measured distribution is normalized by the sum of all peaks accounted for in a fragment:

3 $$ {f}_i={\mathrm{N}}_{\left(\mathrm{M}+\mathrm{i}\right)}/{\displaystyle \sum_{\mathrm{k}=0}^n}{N}_{\left(\mathrm{M}+\mathrm{k}\right)} $$

Such a normalized distribution of m/z peaks (F = {fi}) is then further corrected for naturally occurring isotopes, which is necessary to determine the fractions (m) of artificially labeled isotopomers.

The difference (**D**) between the theoretical (P_t_) and experimental (P_e_) MIDs after the correction of the latter for H+ loss and normalization was further used to characterize sources of errors other than H+ loss.

4 $$ \mathrm{D}=\mathrm{P}\mathrm{e}\hbox{-} \mathrm{P}\mathrm{t} $$

### Calculated and measured MID

**Fig. 1:**
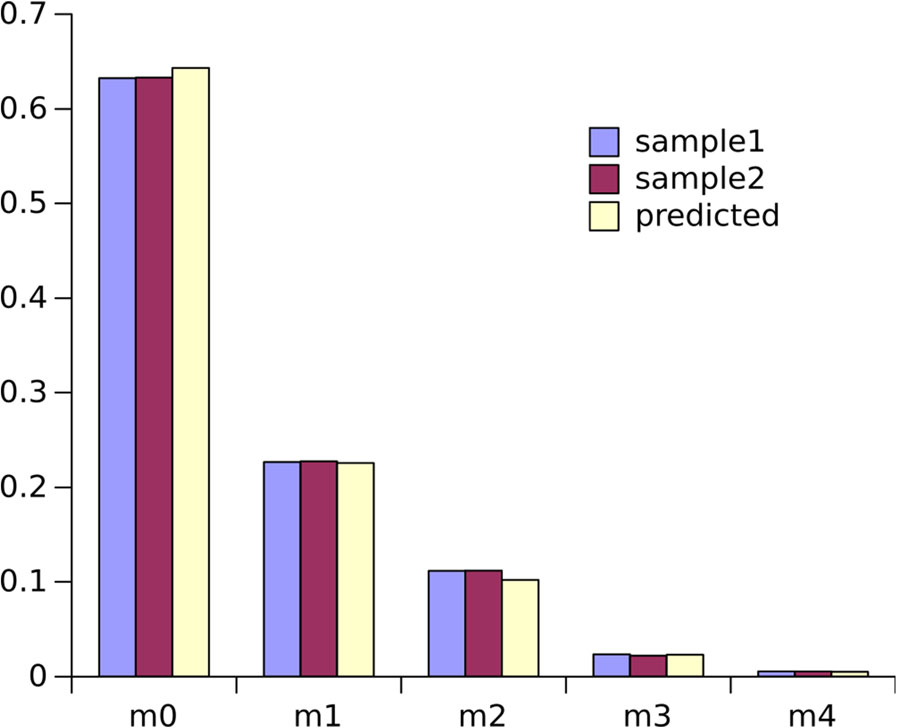
Natural MID, measured for cold TMS-derivative of aspartate. The raw data for two independent samples shown in Table 1 were corrected for the mass shift and normalized (Eqs 1–3). The calculations were performed as described in Additional file 1 Text S1

As an example, the MID for aspartate (peaks from M-1 to M + 5) shown in Table 1 was obtained from a cell culture medium with no ^13^C labeled substrates. The assayed derivative fragment contains 18 C and 3 Si atoms. In theory, such a composition provides 25 possible mass isotopomers (ranging from unlabeled to maximally labeled), but only five mass isotopomers were measurable, as confirmed by calculations indicating that the fractions of isotopomers with the higher mass numbers are vanishingly small. The natural MID, measured and calculated as described in Additional file 1 Text S1, corrected for the mass shift and normalized (Eqs 1-3), is shown in Fig. 1.

The measured MID (from [M] to [M + 4]) was:

$$ \mathrm{P}\mathrm{e}=\left[0.63218,\ 0.22691,\ 0.11163,\ 0.02367,\ 0.00562\right] $$

After the described above correction for the mass shift and normalization (Eqs 1-3) we have the following MID (from [M] to [M + 4]):

5 $$ \mathrm{P}\mathrm{e}=\left[0.6325\ 0.2271\ 0.1118\ 0.0230\ 0.0056\right] $$

The theoretical distribution that provides values for all possible 25 mass isotopomers, is reduced to the size of the experimental value (5), to make them consistent, and renormalized to the sum of the remaining isotopomers:

6 $$ \mathrm{P}\mathrm{t}=\left[0.6435\ 0.2266\ 0.1016\ 0.0232\ 0.0051\right] $$

The difference (**D**) between the calculated and measured spectra was determined for naturally labeled metabolites in the case considered here:

7 $$ \mathbf{D}= P e- P t=\left[-0.01099\kern0.5em 0.00052\kern0.5em 0.01026\kern0.5em -0.00023\ 0.00044\right] $$

The **D**-value for the most abundant mass isotopomer (M), although it is relatively small (~1%), is greater than the difference between the values of direct measurements in various samples normalized by the sum of peaks, as shown for a typical example of a series of technical and biological replicates presented in supplementary Additional file 2 Text S3.

### Obtaining a genuine ^13^C distribution originating from artificially labeled substrates

In general, a measured distribution after being corrected for an H+ loss contains a mixture of naturally occurring isotopomers and those obtained from artificially labeled substrates. A correction for naturally occurring isotopes is necessary to generate a genuine “artificial” ^13^C distribution, i.e., the one originating from substrates artificially enriched with the ^13^C isotope. Here, we briefly describe the method for implementing such a correction in the MIDcor program.

First, the program is used to construct a set of vectors corresponding to the distribution of naturally occurring isotopes in the absence of artificial labels (P0, equal to Pt in Eq. (6)), or to the presence of one (P_1_), two (P_2_), etc., artificial labels in the tested molecules. This set of vectors yields the correction matrix, where the vectors (P_0_), P_1_, …, appear in columns (Table 2).

**Table 2:** A correction matrix for evaluation of “pure” artificial ^13^C distribution

	P_0_	P_1_	P_2_	P_3_	P_4_
m0	0.6435	0	0	0	0
m1	0.2266	0.6468	0	0	0
m2	0.1016	0.2277	0.6623	0	0
m3	0.0232	0.1021	0.2332	0.7396	0
m4	0.0051	0.02333	0.10455	0.26041	1

If, for instance, one artificially labeled carbon is present in the molecule (P_1_), then the probability of finding an unlabeled isotopomer among such molecules is zero. However, in the molecules containing one artificial label, a non-zero likelihood exists of finding more than one ^13^C carbons due to the natural occurrence of the ^13^C isotopes. The other carbons can be ^13^C-labeled following the natural occurrence of ^13^C isotope (renormalized each time to the sum of the remaining isotopomers). In general, for any molecule, containing two, three, etc., artificial labels, the probability of finding fewer labels is virtually zero, and the probability of finding more labels is determined by the natural occurrence of ^13^C isotopes.

To acquire the actual value for artificial labeling, not mixed with naturally occurring isotopes, it is necessary to solve the following equation:

8 $$ \mathbf{m}\times \mathbf{P}=\mathbf{P}\mathbf{e} $$

Here **P** is the matrix presented in Table 2, and **Pe** is the vector shown in Eq. (6).

### The difference between calculated and measured distributions

In the example considered here, aspartate molecules do not contain any artificial ^13^C labels. In this case, the distribution corrected for the number of naturally occurring isotopes should yield a vector **m** not containing any artificial ^13^C labels, i.e., **m** = [1 0 0 0 0]. In fact the solution of Eq. (8) is **m** = [0.9833 0.0064 0.0159–0.0047–0.0002].

The actual solution is different from the one expected, because the theoretical MID calculated for naturally occurring isotopes (Eq. 6) is distinct from the corresponding distribution measured experimentally (Eq. 5) by the vector **D** (Eq. 7). We suggested that such differences between experimental and the corresponding theoretical distributions often arise from the overlap of the mass spectrum of an analyzed metabolite with that of another substance existing in the medium.

### Two ways of correcting measured m/z peaks in the presence of artificial labeling

The correction of the experimental data in the case considered of unlabeled aspartate by subtracting the vector **D** from the experimental data is evident. However, it is not clear how to correct this kind of errors for the tested samples with unknown artificial ^13^C labeling, where the corresponding theoretical spectra cannot be calculated. In samples with unknown artificial ^13^C labeling we correct the error arising from overlapping peaks based on the vector **D** determined for unlabeled samples as described above. In this correction we distinguished two cases: **1**) the difference **D** does not depend on the measured distribution of artificial label in the assayed metabolite, but depends on the composition of the medium and **2**) the difference **D** depends on the distribution of the artificial label in the assayed metabolite, mass distributions of various fragments of the same labeled metabolite probably overlap in the measured range of m/z.

In **case 1** a possible reason for the difference **D** is an overlapping of the analyzed peaks in the mass spectrum with peaks of some compound of the assay medium, which can be the same for labeled as well as for unlabeled samples. The correction consists of subtracting the difference **D** obtained for the unlabeled sample without its modification from the mass spectrum measured for the tested labeled sample (Eq. 4 in general, or Eq. 7 for the case of aspartate in the given experiment).

In **case 2** the difference (Eq. 4) is valid only for the naturally labeled metabolite, and the artificial labeling shifts the difference **D** by the m/z number corresponding to the number of artificially introduced ^13^C atoms. The contribution of any individual artificially labeled mass isotopomer to the modified **D** value should be proportional to the fraction of a particular isotopomer.

Variation of the composition of the assay medium allows for distinguishing between these two causes of inconsistency. To this end, the m/z distribution for the given metabolite not labeled artificially should be measured both (**a**) in the full medium in which the cell was incubated and (**b**) in the solution containing only the tested metabolite and reagents used for the derivatization.

If the evaluated difference **D** (Eq. 4) is greater in the medium (a) than in (b), the cause of the observed discrepancy **D** is overlapping of the analyzed pattern with that for some metabolite of the incubation medium (**case 1**). **D** should be subtracted from the measured normalized spectrum without any change, following Eq. 4, to correct the raw data in this case.

If **D** is equal in both the medium (a) and in (b), the incubation medium does not affect the measurement. In this case, the analyzed mass spectrum probably overlaps with a pattern of another fragment of the same metabolite, which is present in the medium (a) as well as in (b). Therefore the independence of **D** from the medium of incubation characterizes **case 2. D** should be modified considering that labeling with n ^13^C isotopes shifts **D** by n positions to the right and the values of **D** change proportionally to the enrichment in (M + n) isotopomers. Such a modification is implemented as follows.

### Correction of measured m/z distribution (F^0^) for the case in which D depends on the artificial labeling of the assayed metabolite

#### Step 1

Calculate the MID, correcting the measured **Pe** only for H+ loss and for naturally occurring isotopes, as described in Methods.

#### Step 2

Recalculate **D** for the MID obtained by assuming the presence of each artificially introduced ^13^C atom shifts the vector **D**, determined for the unlabeled sample, by one m/z unit and changes proportionally to the intensity of labeling.

#### Step 3

Obtain a new vector **Pe**, by adding the vector **D**, recalculated in **Step 2**, to the experimental distribution following Eq. 4; return to **Step 1**,

The cycle for step 1-step 3 repeats until the vector F and the corresponding distribution of artificially labeled isotopomers **m** stabilizes.

## Results and discussion

### Difference D for some metabolites

The difference between the relative values of corresponding peaks for two technical replicates, calculated based on the data shown in Table 1, is less than 0.03%, and in general, in our laboratory the standard deviation of repeated technical replicates is less than 0.5%. This value characterizes the sensitivity of the method. Table 3 shows the values of the difference **D** (Eq. 8) for some metabolites in the incubation medium (conditions (a)) and the minimal medium containing only the components necessary for derivation (conditions (b)). The obtained deviation from theoretical distribution for most of the tested metabolites in both conditions overcome the sensitivity of the method, as indicated by the values of **D** for various metabolites shown in Table 3. Such a big difference points that there is an additional source of errors that we do not take into consideration.

**Table 3:** The differences (**D**, Eq. 4, expressed as % of total amount of a substance) between calculated and measured MID for some TMS derivatives of metabolites

m/z:	M	M + 1	M + 2	M + 3	M + 4	M + 5	M + 6	Max df,case#
glucose	−0.35	−0.62	0.94	0.02	0.00	0.00	0.00	2.25
+medium	−2.60	0.64	1.60	0.26	0.06	0.01	0.00	case 1
Glu (2–4)	−9.30	7.74	1.33	0.21	nd	nd	nd	−0.10
+medium	−9.20	7.79	1.23	0.22	nd	nd	nd	case 2
Glu (2–5)	−1.30	0.48	0.65	0.04	0.14	nd	nd	0.10
+medium	−1.40	0.51	0.65	0.04	0.18	nd	nd	case 2
Aspartate	−0.98	−0.06	0.88	0.14	0.02	0.00	nd	0.12
+medium	−1.10	0.05	1.03	−0.02	0.04	nd	nd	case 2
serine	−0.70	−0.37	0.87	0.13	nd	nd	nd	0.40
+medium	−1.10	0.02	0.91	0.17	nd	nd	nd	case 2
glycine	−0.50	−0.14	0.57	0.08	0.01	nd	nd	0.70
+medium	−1.20	0.27	0.70	0.23	0.01	nd	nd	case 1

The first row for each metabolite was calculated using Eq. 4 for the measurements in the minimal solution for derivatization (conditions (b), see 3.1.4). The next row indicated as “+medium” was calculated for the measurements in the complete media for cell incubation (conditions (a)). “Glu (2–4)” and “Glu (2–5)” refer to the fragments C2-C4 and C2-C5 of glutamate after ionization in the mass spectrometer, “nd” stands for “not determined”. The right column indicates the maximal difference between the two rows for each metabolite, and, based on this difference and using a threshold value of 0.5%, recommendation to analyze it in accordance with **case 1** or **case 2**

The two measurements for each analyzed metabolite shown in Table 3 are sufficient to verify which one of the two cases determines the value of the correction. In principle, the measurements in a minimal medium can be performed just once, and they can serve for all subsequent experiments analyzed using the specific GC/MS instrument. In this case, only the measurement of the mass spectra of unlabeled metabolites in the specific cell incubation medium is necessary.

As presented in Table 3, both cases considered above probably occur. The discrepancy between the measured and predicted labeling in glucose and glycine increases when passing from the minimal solution (b) to the cell incubation medium (a). This increase is consistent with **case 1**: the m/z values of some components of the medium overlap with the m/z values of a metabolite-of-interest when the artificial labeling is analyzed. Because the composition of the medium does not depend on the labeling of the assayed metabolite, the value **D**, calculated for the unlabeled sample, should be added to the measured vector P_e_ without any change.

On the contrary, in the other cases, the difference does not change if the medium changes. The value of **D** for the glutamate fragment C2–C4 is huge, but it is the same in both media. An unexpected M + 1 peak appears in this fragment. The constant **D** is consistent with **case 2**: the m/z pattern of the assayed fragment overlaps with some other fragment of the same metabolite, such that labeling changes the overlapping patterns. In this case, the above-described correction algorithm should be applied.

If the difference **D** is determined by the composition of the medium, rather than by the artificial labeling of the assayed metabolite, the correction is simple and intuitive. However, in the case in which **D** depends on the artificial labeling of the assayed metabolite, the algorithm for data correction must be validated, and an example of such a validation is described next.

### Validation of the algorithm for correction of the measured MID in the case of natural and uniformly labeled C2-C4 fragments of glutamate

The difference **D** in the case of the C2-C4 fragment of glutamate (Table 2), as concluded above, depends on the artificial labeling of this fragment. Artificial labeling is expected to shift **D** to the right by the number of labeled carbons present in the carbon skeleton. Therefore, it should be corrected using the above-described algorithm. To validate this conclusion, we analyzed the m/z values for spectra measured for artificially labeled commercially available glutamate. The intensities of the peaks corresponding to the mass isotopomers of the C2-C4 fragment of the trifluoroacetamide butyl ester of glutamate are shown in Table 4.

**Table 4:** Intensities of GC/MS peaks in C2-C4 fragment of glutamate for various commercially available isotopomers

m/z	151	152	153	154	155	156	157	158	159
natural	6293	712362	110422	12185	1821	3166	528	8002	877
Q3-^13^C	1562	10455	652415	63645	8745	1225	2767	386	4340
QU-^13^C	315	2406	3923	24427	833038	64103	9910	838	484

Here “Q3-^13^C” states for the sample where only one ^13^C is introduced in position 3, and “QU-^13^C” states for uniformly ^13^C labeled sample

Table 5 demonstrates the distribution of mass isotopomers after the correction for the occurrence of natural isotopes, and normalization. After this correction, a significant but unexpected fraction of the m0 + 1 isotopomer was detected in the unlabeled sample. In the sample containing only one ^13^C atom per molecule, a significant fraction of an unexpected m1 + 1 isotopomer was detected. In the uniformly labeled sample in which all three carbons of the C2–C4 fragment were labeled, the unexpected fraction of m3 + 1 was detected. Indeed, the m/z position of the unexpected isotopomer shifts and is always situated next to the position of the most abundant isotopomer. Such a labeling-dependent shift qualitatively confirms the assumption that the unexpected peak is produced by some derivate of the same labeled metabolite.

**Table 5:** Data shown in Table 4 after a correction for natural isotope occurrence and normalization

	m0	m1	m2	m3	m4
natural	0.90	0.09	0.01	0.0	0.0
Q3-^13^C	0.004	0.935	0.049	0.01	0.001
QU-^13^C	0.003	0.004	0.016	0.927	0.050

The names of the commercial preparations analyzed are the same as in Table 4

However, the value of such unexpected peak for the artificially labeled fragment is not the same as for unlabeled one. The corresponding correction following the correction algorithm resulted in the mass isotopomer distributions shown in Table 6.

**Table 6:** The fully corrected artificial labeling of C2-C4 fragment of glutamate

	m0	m1	m2	m3	m4
natural	1.0	0.0	0.0	0.0	0.0
Q3-^13^C	0.005	1.037	−0.044	0.002	0.
QU-^13^C	0.003	0.004	0.018	1.011	−0.036

After correction for the occurrence of the natural isotope, the algorithm for the correction was applied to the case of the analyzed overlapping pattern with another that depends on the labeling of the studied metabolite.

The quantitative difference between the values of the unexpected peak for the unlabeled metabolite leads to the negative value of approximately 4% for the next-to-the-most abundant mass isotopomer (by mass number). Isotopic effects can explain this quantitative difference from the expected values-as isotopic enrichment increases, the probability of forming an overlapping fragment decreases. Our hypothesis relative to the nature of the isotopic effect is described in the Additional file 3 Text S2. Because this effect is approximately the same for the fragment labeled in just one position, C3, and for the completely labeled fragment, one could conclude that the isotope in the C3 position determines this isotopic effect. Such an isotopic effect was implemented such that **D** determined for the unlabeled substance was applied only for the unlabeled fraction, whereas if it is applied to the labeled fractions, **D** should be multiplied by some factor. This isotopic factor represents a value that, applied to the vector **D**, allows the known labeling in commercial preparations to be reproduced.

Application of such a differential correction with an isotopic factor of approximately 0.6 allows the known labeling of the isotopomers to be reproduced as shown in Table 7. Subsequently, when the isotopic effect is known and considered, the software can be applied to correct the unknown distribution of mass isotopomers. As an example of “unknown” labeling, the mass spectra of various mixtures of commercial samples of unlabeled compounds, and Q3-^13^C and QU-^13^C isotopomers, were measured. The results of this determination were consistent with the prepared mixtures (Table 7).

**Table 7:** Accounting for the isotopic effect in data correction for C2-C4 and C2-C5 fragments of glutamine using the factor of 0.6 for the labeled isotopomers

^13^C-labeling	m0	m1	m2	m3	m4	fragment
natural	1	0	0	0	0	C2C4
natural	1	0	0	0	0	C2C5
3-^13^C-Gln	0.008	0.986	0.0	0.005	0	C2C4
3-^13^C-Gln	0.005	0.9895	0.001	0.003	0.001	C2C5
U-^13^C-Gln	0.004	0.005	0.018	0.973	0	C2C4
U-^13^C-Gln	0.003	0.003	0.01	0.022	0.959	C2C5
n:3:U_90:2:8	0.889	0.020	0.002	0.089	0	C2C4
n:3:U_90:2:8	0.888	0.021	0.001	0.002	0.088	C2C5
n:3:U_40:30:30	0.437	0.277	0.006	0.280	0	C2C4
n:3:U_40:30:30	0.436	0.277	0.003	0.007	0.276	C2C5
n:3:U_95:2:3	0.940	0.026	0.001	0.032	0.0	C2C4
n:3:U_95:2:3	0.940	0.027	0.0	0.001	0.032	C2C5

The calculated artificial labeling of naturally or artificially ^13^C labeled C2–C4 and C2–C5 fragments of glutamine was obtained by applying the algorithm modified to account for the isotopic effect. The samples were prepared either from unlabeled glutamine (natural), ^13^C- glutamine labeled at position 3 (3-^13^C-Gln), uniformly labeled glutamine (U-^13^C-Gln), or various ratios of these samples: natural : 3-^13^C-Gln : U-^13^C-Gln (n:3:U) as indicated

The electron impact ionization procedure used in GC/MS produces various fragments of glutamate (e.g., a fragment, C2–C5 in addition to C2–C4). The values of **D** corresponding to the C2–C5 fragment are much less than those for the C2–C4 fragment (Table 3). Mass distribution measurements are also used to determine the glutamate labeling. The fractions determined based on the mass distribution of the C2–C5 fragment are also presented in Table 7. The corrections applied produce the same results for both fragments, although the deviation of the measured from the expected mass isotopomer distribution (vector **D**) for the C2–C4 fragment is much larger than that for the C2–C5 fragment. Thus, the applied algorithm yields the correct fractions of the mixtures of commercial glutamine preparations based on the mass distribution either in fragment C2–C4, characterized by large values of **D**, or characterized by a relatively small value for **D** in the C2-C5 fragment.

Thus, the validation of our algorithm for the correction of overlapping peaks for Glu fragment C2–C4 revealed that although the artificial labeling shifts the vector **D** as in **case 2**, it also changes the values of **D** proportionally. Because the change in **D** depends on the isotopic composition we termed it an “isotopic effect.” We did not study here the actual nature of this effect; this term simply represents the change in **D** determined by artificial labeling. The isotopic effect can be quantified by a factor that, being multiplied by **D**, allows the measured MID to be reproduced in the presence of artificial labeling. Once determined by fitting a sample with a priori known artificial labeling, it can be validated by the calculation of labeling of other known samples using the algorithm described here, and the factor determined. Here, we determined the value of the “isotopic” factor as 0.6 by fitting the known MID in the C2–C4 fragment of commercial 3-^13^C-Gln (Table 7). Subsequently, we validated this factor by calculating the MID in U-^13^C-Gln and in three other samples presented in Table 7. The agreement of the calculations with the known MID, as well as with the results of analysis of another fragment, C2–C5, allows us to conclude that the applied method of correction is valid, although the actual nature of this effect should be further investigated.

The considered here examples show that more than one substance can commonly have mass spectra in the same m/z region. Such overlapping of an analyzed metabolite mass spectrum with that of another substance hides the actual distribution of isotopes, originating from artificially ^13^C enriched substrates. Our computer program MIDcor corrects the errors arising from such an overlapping. It implements an algorithm that supports two cases of correction based on **D**, obtained in two different media-in a full cell incubation medium, and in a minimal medium prepared with only the assayed metabolite and reactants for derivation. If some values of the vector **D** obtained in the complete medium are larger than the corresponding values of **D** for the minimal medium by > 0.5%, then, the MIDcor program applies **case 1**: subtracting the non-modified **D** from the normalized spectra of samples designed for testing before their correction for naturally occurring isotopes. If **D** is the same in both media, then the MIDcor program applies the algorithm appropriate for **case 2**: modifying **D** by shifting it to the right on the m/z scale by the number of artificial isotopes present and changing it proportionally to the fractions of the corresponding mass isotopomers.

In **case 2**, the correction, applied to a sample with a priori known artificial labeling allows determining the isotopic effect, defined as a change of **D** values induced by substitution of some of the ^12^C atoms in the carbon skeleton of a molecule by ^13^C atoms.

## Conclusions

The MIDcor program reveals the distribution of ^13^C mass isotopomers originating exclusively from artificially ^13^C enriched substrates. It corrects the raw mass spectrum of a considered metabolite (1) for the occurrence of the natural isotopes, and (2) for possible overlapping with mass spectra of other substances. Whereas the former is a standard procedure implemented in various computer programs, the latter is specific to our algorithm. To correct the peaks overlapping MIDcor calculates the theoretical mass isotopomer distribution for the case of only naturally occurring isotopes. Then it finds differences (**D**) of the calculated distribution from that measured without artificial labeling either in the complete medium of cell incubation or in minimal medium containing only products of the metabolite derivatization. Values in vector **D** > 1% are considered to indicate overlapping peaks. If **D** is significantly greater in full than in minimal medium, we conclude that the considered spectrum overlaps with that of another substance presented only in full medium. If **D** is similar in both media, the considered spectrum overlaps with that of another derivate of the studied metabolite, presented in both media. Based on the determined **D**, MIDcor corrects the peaks overlapping in any artificially ^13^C labeled samples, treating these two cases differently. If the metabolite labeling changes the probability of peaks overlapping, a spectrum of the metabolite with a priori known artificial labeling should be fit. The usage instructions together with the code can be found at https://github.com/seliv55/mid_correct.

## Additional files

Additional file 1: Text S1.pdf. Calculation of natural ^13^C distribution. (PDF 58 kb)
Additional file 2: Text S3.pdf. An example of standard deviations in a series of technical and biological replicates. (PDF 65 kb)
Additional file 3: Text S2.pdf. Proposed fragment, which overlap with glutamate C2-C4 in GC/MS spectra. (PDF 926 kb)

## Abbreviations

D: The difference between the theoretical and experimental MID after the correction of the latter for H+ loss and normalization
FBS: Fetal bovine serum
GC/MS: Gas chromatography coupled to mass spectrometry
Gln: Glutamine
Glu: Glutamate
m/z: Mass/charge
MID: Mass isotopomer distribution
MS: Mass spectrometry
TMS: Tetramethylsilane
