# Supplementary material for: MIDcor, an R-program for deciphering mass interferences in mass spectra of metabolites enriched in stable isotopes
Source: BMC Bioinformatics. 2017 Feb 3;18:88. doi: 10.1186/s12859-017-1513-3 (PMC5291980; doi:10.1186/s12859-017-1513-3)
Supplement: Additional file 1: — Text S1.pdf. Calculation of natural 13C distribution. (PDF 58 kb) [file 12859_2017_1513_MOESM1_ESM.pdf]

Text S1

Calculation of natural ^{13}C distribution

Natural abundance of ^{13}C is about 1.1% (P_{13C}~1.1%). The probability to find I number of ^{13}C atoms in fixed positions p in a molecule, containing in total n carbons, is

$$P(i,n,p)= (P_{13C})^{i}\times(P_{12C})^{(n-i)} \quad (2.1)$$

The number of various combinations of positions for i ^{13}C and (n-i) ^{12}C atoms is:

$$K(i,n) = n!/(i!\times(n-i)!) \quad (2.2)$$

Each of K fixed positions has the same probability P(i,n,p). Since mass spectrometry does not distinguish between the positions of labeled atoms inside the molecule, we are interested only in total probability of finding i ^{13}C atoms in a n-carbon molecule:

$$P_{(M+i)}(n) = (P_{13C})^{i}\times(P_{12C})^{(n-i)} \times n!/(i!\times(n-i)!) \quad (2.3)$$

Here M is the mass number of lightest isotopomer and i is an integer between 0 and n ($0 \le i \le n$).

If in the assayed fragment various isotopes of other atoms are present, this can be taken into consideration. Let us consider an example of the presence of one or several Si, which, in addition to the main isotope ^{28}Si with probability P_{Si0}~0.9223, contains essential amounts of two other isotopes ^{29}Si and ^{30}Si with probabilities P_{Si1}~0.0467 and P_{Si2}~0.031 correspondingly.

In the initial step the algorithm calculates $P_{(M+i)}(n)$ for the case of the presence of only n carbon atoms (Eq. 2.3). Then this distribution is recalculated to account for one more Si atom as follows:

The lightest mass isotopomer ($P_M(n,Si)$ contains only lightest carbon and silicium isotopes:

$$P_M(n,Si)=P_{Si0}\times P_M(n) \quad (2.4)$$

The (M+1) mass isotopomer contains either only lightest C isotopes and ^{29}Si, or one ^{13}C and ^{28}Si:

$$P_{(M+1)}(n,Si)=P_{Si1}\times P_M(n)+P_{Si0}\times P_{(M+1)}(n) \quad (2.5)$$

The probabilities of finding heavier isotopomers are calculated similarly, but accounting for the third probability that they also can contain isotope ^{30}Si:

$$P_{(M+i)}(n,Si)=P_{Si2}\times P_{(M+i-2)}(n)+P_{Si1}\times P_{(M+i-1)}(n)+P_{Si0}\times P_{(M+i)}(n) \quad (2.6)$$

The presence of one Si provides two more mass isotopomers, $P_{(M+n+1)}(n,Si)$ and $P_{(M+n+2)}(n,Si)$.

In the case of the presence of more than one Si atoms in the assayed fragment, to calculate the probabilities changed by the presence of second and subsequent Si atoms, the program returns to step i, but with the following changes. The values $P_{(M+i)}(n,Si)$, already calculated in Eqs 2.4-2.6, are put into the right hand side of these equations as $P_{(M+i)}(n)$, and n increased by 2 in accordance with the addition introduced by previously accounted Si.

The above described algorithm can be applied similarly to account for other isotopes that the assayed fragment may contain.
