# Supplementary material for: MIDcor, an R-program for deciphering mass interferences in mass spectra of metabolites enriched in stable isotopes
Source: BMC Bioinformatics. 2017 Feb 3;18:88. doi: 10.1186/s12859-017-1513-3 (PMC5291980; doi:10.1186/s12859-017-1513-3)
Supplement: Additional file 2: — Text S3.pdf. An example of standard deviations in a series of technical and biological replicates. (PDF 65 kb) [file 12859_2017_1513_MOESM2_ESM.pdf]

Text S3

An example of standard deviations in a series of technical and biological replicates.

An example of experimentally measured mass isotopomer spectra with corrected baseline is shown in Table S1.

Table S1. Intensities of peaks in m/z spectra corresponding to the mass isotopomers of ribose-5-phospate

Sample name	M-1	M	M+1	M+2	M+3	M+4	M+5	M+6
RibNor1_01	3974	1063984	272009	143895	38544	23614	2243	990
RibNor1_02	3119	721679	182976	97154	25424	14900	1578	609
RibNor1_03	2609	732113	187751	95813	25924	14795	1999	508
RibNor2_01	2561	700110	185168	94800	25559	14905	1766	684
RibNor2_02	2166	542016	137917	73172	19521	11879	1162	595
RibNor2_03	2801	894353	232028	123178	32118	18454	2116	1010
RibNor3_01	2585	662346	171763	92918	24804	14428	1914	344
RibNor3_02	2242	788305	207194	109892	30734	16526	1825	497

Here the column M shows the intensities of the lightest isotopomer (257 in the presented example) obtained for several replicates. (M+i) designates heavier mass isotopomers. (M-1) designates intensities of lighter mass isotopomer appeared due to electron impact in the mass spectrometer. The first digit in the sample name indicates the biological replicate (plate with growing cells), and the last digit indicates the technical replicates (injections of the solution prepared from the same plate).

Table S2 represents the data of Table S1 normalized by the sum of all peaks and the standard deviation calculated for the technical replicates of the same biological sample and the whole set of samples. It shows that even in the latter case the maximal standard deviation is less than 0.35%.

Table S3, which presents the same data after their full correction for the natural isotope occurrence and for the peaks overlapping, shows that the correction procedures practically do not increase the standard deviation of technical as well as biological replicates.

Table S2. Normalized intensities of peaks presented in Table S1, expressed as % of the sum of ions from all peaks in the corresponding spectrum, standard deviations, and correction factor.

Sample	M-1	M	M+1	M+2	M+3	M+4	M+5	M+6
RibNor1_01	0.257	68.7	17.6	9.29	2.49	1.52	0.145	0.0639
RibNor1_02	0.298	68.9	17.5	9.28	2.43	1.42	0.151	0.0581
RibNor1_03	0.246	69	17.7	9.03	2.44	1.39	0.188	0.0479
SD	0.0274	0.152	0.11	0.148	0.0316	0.0685	0.0236	0.0081
RibNor2_01	0.25	68.3	18.1	9.24	2.49	1.45	0.172	0.0667
RibNor2_02	0.275	68.7	17.5	9.28	2.48	1.51	0.147	0.0755
RibNor2_03	0.214	68.5	17.8	9.43	2.46	1.41	0.162	0.0773
SD	0.0303	0.241	0.281	0.0993	0.0165	0.047	0.0125	0.00568
RibNor3_01	0.266	68.2	17.7	9.57	2.55	1.49	0.197	0.0354
RibNor3_02	0.194	68.1	17.9	9.5	2.66	1.43	0.158	0.0429
SD	0.0512	0.06	0.154	0.051	0.0719	0.0408	0.0279	0.00532
SD global	0.033	0.325	0.203	0.17	0.074	0.0473	0.0193	0.0152

Standard deviations (SD and SD global) are calculated for the technical replicates and for the global set of samples that unites the technical and biological replicates (n=8).The designations of the columns and rows are the same as in Table S1.

Table S3. Fully corrected mass isotopomer distributions (mole percent) calculated for each technical replicate from the data presented in Table S2, and standard deviations.

Sample	M	M+1	M+2	M+3	M+4	M+5
RibNor1_01	79.2	10.5	7.26	1.76	1.29	0.016
RibNor1_02	79.5	10.4	7.26	1.69	1.18	0.029
RibNor1_03	79.5	10.6	6.95	1.74	1.15	0.069
SD	0.171	0.115	0.178	0.0332	0.0719	0.0276

RibNor2_01	78.7	11.1	7.14	1.77	1.21	0.049
RibNor2_02	79.3	10.4	7.26	1.74	1.27	0.02
RibNor2_03	78.9	10.8	7.39	1.71	1.17	0.041
SD	0.288	0.348	0.122	0.0296	0.0503	0.015
RibNor3_01	78.6	10.7	7.55	1.8	1.24	0.071
RibNor3_02	78.5	11	7.43	1.92	1.17	0.037
SD	0.105	0.176	0.08	0.0848	0.0516	0.024
SD global	0.387	0.26	0.183	0.0705	0.0512	0.0206
D	−1.196	−0.381	1.227	0.159	0.162	0.030

The presented data are those given in Table S2, corrected for naturally occurring isotopes and for peaks overlapping. The latter was performed based on the difference "**D**" between the really measured and theoretical distribution calculated for the naturally labeled sample as explained in the main text. This difference is shown in the last row. Standard deviations (SD and SD global) are calculated respectively for the technical replicates and for the global set of samples that unites the technical and biological replicates (n=8). The designations of the columns and rows are the same as in Table S1.

The last row in Table S3 shows the difference between the really measured and theoretical distribution calculated for the naturally labeled sample. Its maximal absolute value is much higher than the SD of all technical replicates as well as the SD for the whole set of samples.
