# Supplementary material for: MIDcor, an R-program for deciphering mass interferences in mass spectra of metabolites enriched in stable isotopes
Source: BMC Bioinformatics. 2017 Feb 3;18:88. doi: 10.1186/s12859-017-1513-3 (PMC5291980; doi:10.1186/s12859-017-1513-3)
Supplement: Additional file 3: — Text S2.pdf. Proposed fragment, which overlap with glutamate C2-C4 in GC/MS spectra. (PDF 926 kb) [file 12859_2017_1513_MOESM3_ESM.pdf]

Text S2

Proposed fragment, which overlap with glutamate C2-C4 in GC/MS spectra.

In the main text the general possibility of overlapping glutamate C2-C4 fragment with some fragment, which could be labeled as well as the assayed glutamate, is considered. We offer here a hypothetical mechanism of formation of such a fragment.

The derivate of glutamate used for GC/MS analysis is shown in Fig.S1. Electronic impact (EI) splits the molecules of derivates. In particular the fragment C2-C4 is formed by splitting the whole molecule

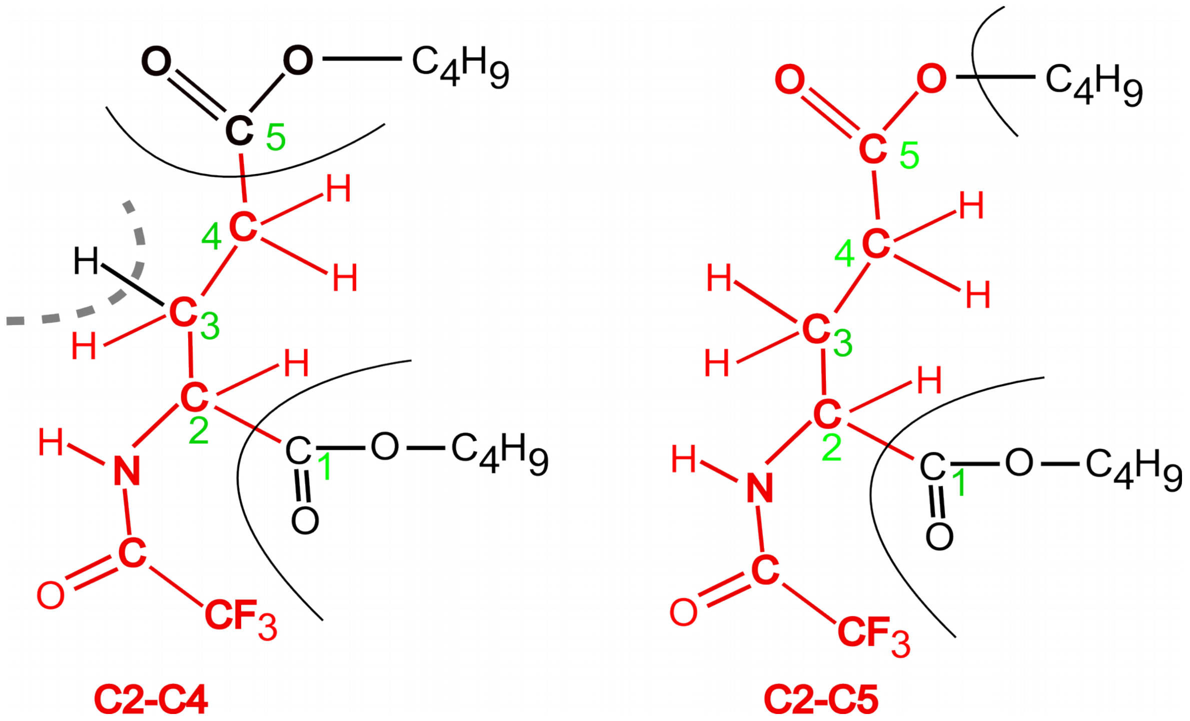

and liberation of two identical fragments containing C5 and C1 atoms of glutamate (left panel).

Figure S1. A hypothetical mechanism of formation of two fragments of glutamate C2-C4 that differ by 1 unit in mass. The numeration of carbons of original glutamate molecule is shown in green. Solid black curves indicate the fragments of the derivate of glutamate (shown in black), liberation of which due to electronic impact (EI) results in the formation of the sought fragments (shown in red). Left panel, the formation of the fragment C2-C4, which may or may not contain the proton (separated by dashed thick line). Right panel, the formation of C2-C5 fragment.

The molecular weight of this fragment (152) assumes also a liberation of one proton, which could be a proton connected with C3 atom (separated by dashed thick line in the left panel of Fig.S1). However, the probability of liberation of a proton is less than 100% and some fragments can contain this proton. Such a fragment is one unit of mass heavier than the most abundant C2-C4 fragment and its GC/MS pattern would always be shifted by one unit whether the glutamate molecule is labeled by 13C or not. Overlapping the patterns of these two fragments would result in the sum, which is different from theoretical prediction calculated without accounting such an overlapping, as described in the main text.

The probability of losing the proton linked to C3 could be different whether C3 is ^{12}C or ^{13}C isotope. This explains the failure to correct such an overlapping for labeled glutamate based only on the data obtained for unlabeled glutamate. We suppose that C3 is the only position where the proton can be lost, because the amplitude of the additional pattern is the practically the same for glutamate labeled in C3 and for uniformly labeled glutamate (although shifted by different m/z value).

In the fragment C2-C5 (right panel) all the protons are stable, but the formed anion binds a proton, as can be deduced from the molecular weight of this fragment (198). Such a binding has a low probability and evidently isotope-independent.
